# Subdivision of de-novo metastatic nasopharyngeal carcinoma based on tumor burden and pretreatment EBV DNA for therapeutic guidance of locoregional radiotherapy

**DOI:** 10.1186/s12885-021-08246-0

**Published:** 2021-05-11

**Authors:** Jin-Hao Yang, Xue-Song Sun, Bei-Bei Xiao, Li-Ting Liu, Shan-Shan Guo, Jia-Dong Liang, Guo-Dong Jia, Lin-Quan Tang, Qiu-Yan Chen, Hai-Qiang Mai

**Affiliations:** 1 Sun Yat-sen University Cancer Center, State Key Laboratory of Oncology in South China, Collaborative Innovation Center for Cancer Medicine, Guangdong Key Laboratory of Nasopharyngeal Carcinoma Diagnosis and Therapy, 651 Dongfeng Road East, Guangzhou, 510060 People’s Republic of China; 2 Department of Nasopharyngeal Carcinoma, Sun Yat-sen University Cancer Center, 651 Dongfeng Road East, Guangzhou, 510060 People’s Republic of China; 3 Department of Thyroid and Breast Surgery, The First Affiliated Hospital, Sun Yat-sen University, Guangzhou, 510000 China

**Keywords:** Nasopharyngeal carcinoma, Distant metastasis, Locoregional radiotherapy, EBV DNA, Risk stratifications

## Abstract

**Background:**

Nasopharyngeal carcinoma (NPC) is a malignancy predominantly associated with infection by the Epstein-Barr virus (EBV). Approximately 12,900 new cases of NPC occur each year, with more than 70% of cases occurring in the east and southeast Asia. NPC is different from ordinary head and neck squamous cell carcinoma due to its particular biological properties and it is highly sensitive to radiotherapy. With the development of RT technology, the 3-year local control rate and survival rates of non-metastatic NPC reached 80–90% in the intensity-modulated RT (IMRT) era. However, whether distant metastatic NPC (de novo mNPC, dmNPC) should receive locoregional RT (LRRT) needs to be clarified.

**Results:**

Multivariate analysis identified three independent prognostic factors: Epstein-Barr virus (EBV) DNA, number of metastatic lesions, and number of metastatic organs. Through these factors, all patients were successfully divided into 3 subgroups: low-risk (single metastatic organ, EBV DNA ≤ 25,000 copies/ml, and ≤ 5 metastatic lesions), intermediate-risk (single metastatic organ, EBV DNA > 25,000 copies/ml, and ≤ 5 metastatic lesions), and high-risk (multiple metastatic organs or > 5 metastatic lesions or both). By comparing LRRT and non-LRRT groups, statistical differences were found in OS in the low-risk and intermediate-risk subgroups (*p* = 0.039 and *p* = 0.010, respectively) but no significant difference was found in OS in the high-risk subgroup (*p* = 0.076). Further multivariate analysis of different risk stratifications revealed that LRRT can improve OS of low- and intermediate-risk subgroups.

**Conclusions:**

The risk stratification of dmNPC may be used as a new prognostic factor to help clinicians organize individualized LRRT treatment to improve the survival outcomes of dmNPC patients.

**Supplementary Information:**

The online version contains supplementary material available at 10.1186/s12885-021-08246-0.

## Introduction

Nasopharyngeal carcinoma (NPC) is a malignancy predominantly associated with infection by the Epstein-Barr virus (EBV) [1]. It originates in the nasopharyngeal epithelium, which is found on the nasopharyngeal posterior wall. Approximately 12,900 new cases of NPC occur each year, with more than 70% of cases occurring in the east and southeast Asia [1, 2]. NPC is different from ordinary head and neck squamous cell carcinoma due to its particular biological properties and it is highly sensitive to radiotherapy [2]. With the development of radiotherapy technology, the 3-year local control rate and survival rates of non-metastatic NPC reached 80–90% in the intensity-modulated radiotherapy (IMRT) era [3]. Notably, among new cases of NPC, 6–15% of patients are diagnosed with distant metastatic NPC (de-novo mNPC, dmNPC) [4, 5]. Whether these patients should receive LRRT needs to be clarified.

According to a previous study, dmNPC patients that underwent LRRT plus palliative chemotherapy (PCT) achieved greater overall survival (OS) than those that received PCT alone [6]. However, it was not clear whether all patients benefited from the primary tumor treatment [6]. In 2020, the National Comprehensive Cancer Network (NCCN) guidelines recommended LRRT alone following systemic chemotherapy for patients with oligometastatic disease [7]. Nevertheless, the numbers of metastatic sites and organs that should be considered as “localized” or “widespread” have not been reported. Additionally, the previous studies rarely analyze pretreatment plasma EBV DNA concentration, which is regarded as a prognosis indicator for NPC, to select candidates for LRRT [6].

Therefore, this study is conducted to investigate prognostic factors for dmNPC patients and to identify patients who achieved improved OS after LRRT by taking the prognostic factors into consideration. The aim of the study was to provide important information for the individualized treatment of these patients.

## Methods

All methods were carried out in accordance with relevant guidelines and regulations as the National Comprehensive Cancer Network (NCCN) guidelines and eighth edition of the American Joint Committee on Cancer staging system. All patients in the study were staged by eighth edition of the American Joint Committee on Cancer staging system and all patients were treated according to the NCCN guidelines.

### Patients and diagnosis

**Fig. 1:**
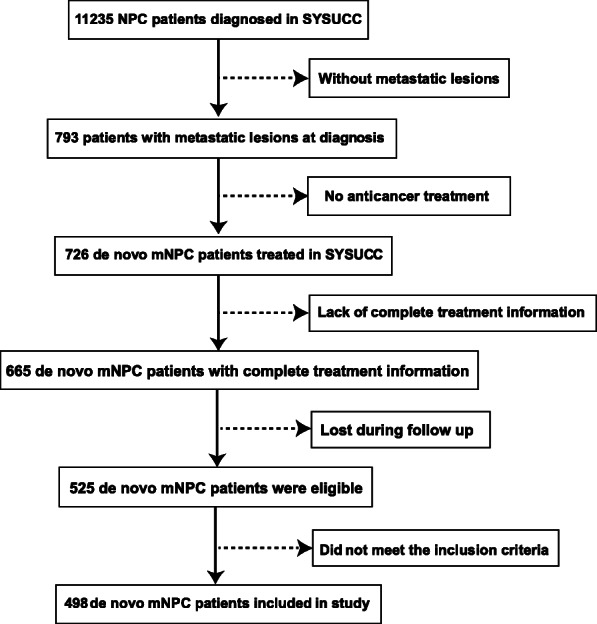
Flow chart showing patients enrollment in study cohort

In total, 11,235 patients were newly diagnosed with NPC in the Sun Yat-sen University Cancer Center in China from November 2006 to October 2016. Of these, 498 patients were enrolled in this retrospective study (Fig. 1) according to the following inclusion criteria: (I) primary lesions in nasopharyngeal were diagnosed histologically and metastatic lesions of dmNPC were diagnosed radiologically or histologically; (II) age between 18 and 70 years; (III) no history of malignancy or synchronous cancer; (IV) treatment with cisplatin-based chemotherapy regimen; (V) normal hematopoietic function: white blood cell count ≥4 × 10^9^/L, platelets ≥100 × 10^9^/L, hemoglobin ≥90 g/L, and neutrophil granulocytes > 2.0 × 10/L; (VI) normal liver function test: aspartate aminotransferase and alanine aminotransferase < 2.5-fold of upper limit of normal (ULN), and total bilirubin < 2.0 × ULN; (VII) normal renal function test: creatinine clearance ≥60 ml/min or creatinine ≤1.5 × ULN; and (VIII) male or non-pregnant female. All patients were restaged based on the 8th edition of the American Joint Committee on Cancer/International Union Against Cancer staging system. This study was approved by the clinical research ethics committee of Sun-Yat sen university cancer center, and written informed consent was obtained from each patient.

During enrollment, all patients in the cohort received primary lesion biopsy under nasal endoscopy. Needle biopsy under CT guidance was performed for some metastatic organs such as liver and lung when the positions and volumes of metastatic lesions were suitable to perform it. Because it was difficult to perform biopsy for metastatic lesions of bones, biopsy on bones was not performed. General evaluation tests mainly included physical examination of the head and neck region (including nasopharynx and cervical lymph nodes), physical examination of the nervous system, EBV serologic tests, EBV DNA quantitative determination, nasal endoscopy, lesion biopsy, head and neck magnetic resonance imaging (MRI) scan, emission compared tomography (ECT) scan, and chest and abdominal CT scan. Positron emission tomography (PET-CT) was considered an optional evaluation test based on the patient’s financial burden.

Metastatic lesions and organs were evaluated based on radiological criteria. The number of metastatic bone lesions and metastatic lesions (excluding bones) were assessed by ECT (or PET-CT) and chest and abdominal CT (or PET-CT) scans, respectively. The number of metastatic organs was evaluated by both ECT and chest and abdominal CT (or PET-CT) scans.

### Plasma EBV DNA essay

Pretreatment plasma EBV DNA concentrations were measured using quantitative polymerase chain reaction. The detailed procedure is reported elsewhere [8].

### Treatment

All included patients received cisplatin-based induction chemotherapy (IC). The common PCT regimens were as follows: (I) triplet docetaxel–cisplatin–fluorouracil: 60 mg/m^2^ of docetaxel and 60 mg/m^2^ of cisplatin on day 1 plus 500–800 mg/m^2^ of 5-fluorouracil for 120 h; (II) docetaxel–cisplatin: 75 mg/m^2^ of docetaxel on day 1 plus 20–25 mg/m^2^ of cisplatin on days 1–3; (III) cisplatin–fluorouracil: 20–25 mg/m^2^ of cisplatin on days 1–3 plus 800–1000 mg/m^2^ of 5- fluorouracil for 96 h; (IV) gemcitabine–cisplatin: 1000 mg/m^2^ of gemcitabine on day 1 plus 20–25 mg/m^2^ of cisplatin on days 1–3. Each regimen was administered intravenously every 3 weeks for a total of 4–6 courses. Among the 498 dmNPC patients, 311 received LRRT after IC with two-dimensional conventional radiotherapy (2DCRT) or IMRT techniques. There were 92 patients receiving 2DCRT and 219 patients receiving IMRT. The total radiation doses were 68–70 Gy for nasopharyngeal and neck lesions, with a frequency of five fractions per week divided in 1.8–2.2 Gy fractions [9].

### Follow-up

All patients underwent follow-up examinations every 3 months for the first 3 years and every 6 months thereafter. The examinations included EBV DNA copy detection, nasopharyngoscopy, head and neck MRI scan, chest and abdominal CT scan, and ECT or PET/CT scans. The primary endpoint of this study was OS, which was measured from initial diagnosis to death from any cause or loss to follow-up.

### Statistical analysis

The clinical characteristics of patients from different treatment groups were compared using the Pearson χ^2^ test. The number of metastatic lesions and EBV DNA copies were transformed into dichotomous variables based on cutoff values defined by the receiver-operating characteristic (ROC) analysis. The survival outcomes of patients from different subgroups were analyzed using Kaplan-Meier curves and the log-rank test. The independent prognosis predictors were evaluated using the Cox proportional hazards regression model. All data analyses were performed using the Statistical Package for Social Sciences (SPSS for macOS, version 21.0, IBM Corp., Armonk, NY). A two-tailed *p* < 0.05 was considered statistically significant.

## Results

### Patient characteristics

Among them, 375 (75.3%) and 123 (24.7%) patients had single and multiple metastatic organs, respectively. The median age was 47 years (range, 18–77 years) and most patients were male (83.1%, 414/498). Regarding metastatic lesions, 338 (67.9%) and 160 (32.1%) patients had five or less and more than five lesions, respectively. Based on ROC analysis, the EBV DNA copies cutoff value was set at 25,000 copies/ml and 284 (57.0%) patients had levels that surpassed this value. As shown in Table 1, statistical differences were found in the number of metastatic organs, number of metastatic lesions, and pretreatment EBV DNA copies between the different treatment groups.

**Table 1:** The clinical characteristics of the patients that did RT and did not do RT

Characteristic	Total	Non-LRRT	LRRT	*P* value
	N (%)	N (%)	N (%)	
**Age (years)**				
≤ 47	246 (49.4)	87 (46.5%)	159 (51.1%)	0.355
> 47	252 (50.6)	100 (53.5%)	152 (48.9%)	
**Gender**				
Male	414 (83.1)	155 (82.9%)	259 (83.3%)	1.000
Female	84 (16.9)	32 (17.1%)	52 (16.7%)	
**Smoking**				
No smoking	278 (55.8)	109 (58.3%)	169 (54.3%)	0.403
Smoking	220 (44.2)	78 (41.7%)	142 (45.7%)	
**Family history**				
No	447 (89.8)	170 (90.9%)	277 (89.1%)	0.545
Yes	51 (10.2)	17 (9.1%)	34 (10.9%)	
**T stage**				
T_1_-T_2_	83 (16.7)	29 (15.5%)	54 (17.4%)	0.621
T_3_-T_4_	415 (83.3)	158 (84.5%)	257 (82.6%)	
**N stage**				
N_0_-N_1_	103 (20.7)	30 (16.0%)	73 (23.5%)	0.052
N_2_-N_3_	395 (79.3)	157 (84.0%)	238 (76.5%)	
**No. of metastatic organs**				
1	375 (75.3)	116 (62.0%)	259 (83.3%)	< 0.001
> 1	123 (24.7)	71 (38.0%)	52 (16.7%)	
**No. of metastatic lesions**				
≤ 5	338 (67.9)	97 (51.9%)	241 (77.5%)	< 0.001
> 5	160 (32.1)	90 (48.1%)	70 (22.5%)	
**EBV-DNA (Copies/ml)**				
≤ 25,000	214 (43.0)	59 (31.6%)	155 (49.8%)	< 0.001
> 25,000	284 (57.0)	128 (68.4%)	156 (50.2%)	
**Chemotherapy regimens**				
TPF	128 (25.7)	40 (21.4%)	88 (28.3%)	0.001
TP	121 (24.3)	36 (19.3%)	85 (27.3%)	
PF	129 (25.9)	58 (31.0%)	71 (22.8%)	
GP	27 (5.4)	18 (9.6%)	9 (2.9%)	
Others	93 (18.7)	35 (18.7%)	58 (18.6%)	

### Analysis of clinical characteristics’ influences on the prognosis of dmNPC patients

**Fig. 2:**
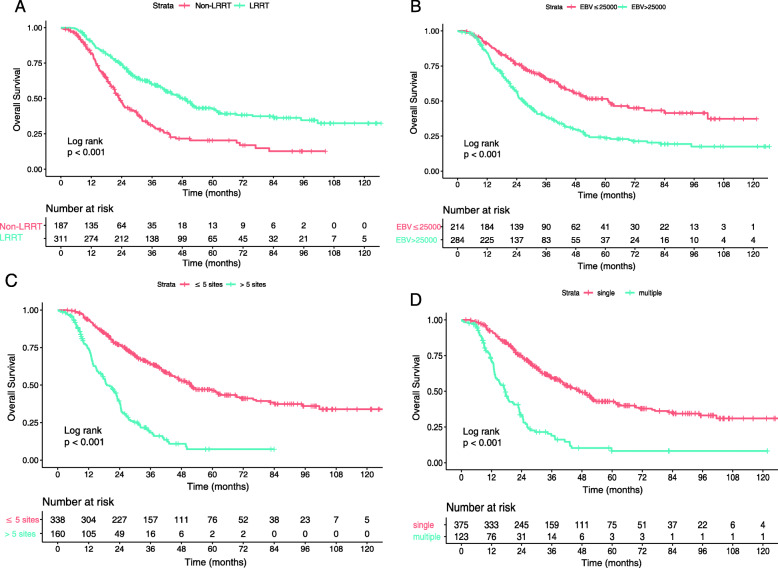
Kaplan–Meier survival curves for the factors that may influence survival outcomes in the training cohort. Radiotherapy (**a**), Epstein-Barr virus DNA copies (**b**), numbers of metastatic lesions (**c**), numbers of metastatic organs (**d**)

**Fig. 3:**
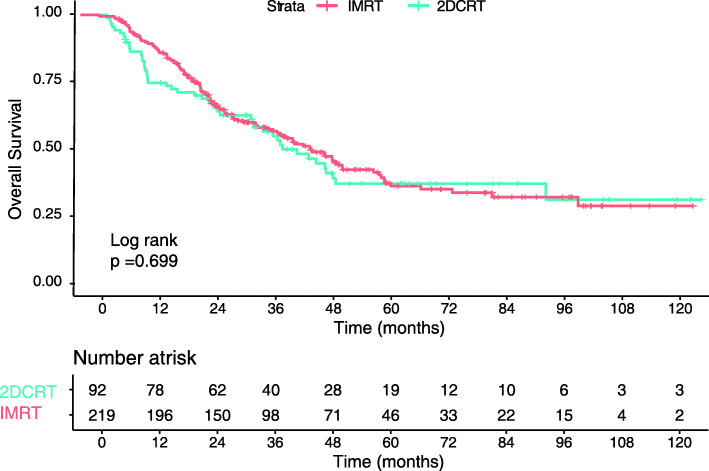
Kaplan–Meier survival curves for 2DCRT and IMRT

All factors that may influence prognosis were included in the Cox proportional hazards regression model. As shown in Table 2, there was a higher mortality risk for patients who had metastasis in multiple organs (hazard ratio [HR], 1.897; 95% confidence interval [CI], 1.401–2.568; *p* < 0.001), more than five metastatic lesions (HR, 2.246; 95% CI, 1.670–3.020; *p* < 0.001), or pretreatment EBV DNA concentrations above 25,000 copies/ml (HR, 1.479; 95% CI, 1.132–1.930; *p* = 0.004), whereas patients who underwent LRRT had a lower risk of death (HR, 0.665; 95% CI, 0.511–0.864; *p* = 0.002). Thus, it could be concluded that multiple organs metastasis, over five metastatic lesions, and EBV DNA concentration above the cutoff value may represent risk factors. The addition of LRRT to PCT may reduce risk of death for dmNPC. The Kaplan-Meier survival curves also showed an association between LRRT and improved OS (3-year OS, 27% vs. 13%; *p* < 0.001) (Fig. 2a). However, compared with 2DCRT, IMRT might not improve OS of dmNPC (Fig. 3). Multivariable analysis (Table 3) also showed that the radiotherapy techniques were not an independent diagnostic factor. Obviously, patients with the aforementioned risk factors had shorter OS than other patients (*p* < 0.001 for all) (Fig. 2b-d).

**Table 2:** Multivariable analysis for patients prognosis

Characteristic	Hazard ratio	95%CI	*P* value
**Age (years)**			
≤ 47	Reference		
> 47	1.183	0.929–1.507	0.172
**Gender**			
Male	Reference		
Female	0.927	0.669–1.285	0.650
**Smoking**			
No smoking	Reference		
Smoking	1.160	0.903–1.489	0.246
**Family history**			
No	Reference		
Yes	0.746	0.469–1.187	0.217
**T stage**			
T_1_-T_2_	Reference		
T_3_-T_4_	0.940	0.686–1.289	0.702
**N stage**			
N_0_-N_1_	Reference		
N_2_-N_3_	1.379	0.982–1.886	0.056
**No. of metastatic organs**			
1	Reference		
> 1	1.897	1.401–2.568	< 0.001
**No. of metastatic lesions**			
≤ 5	Reference		
> 5	2.246	1.670–3.020	< 0.001
**EBV-DNA (Copies/ml)**			
≤ 25,000	Reference		
> 25,000	1.479	1.132–1.930	0.004
**Chemotherapy regimens**			
TPF	Reference		
TP	0.799	0.560–1.141	0.218
PF	0.835	0.580–1.203	0.334
GP	0.881	0.619–1.254	0.481
Others	0.821	0.460–1.464	0.504
**LRRT**			
Non-LRRT	Reference		
LRRT	0.665	0.511–0.864	0.002

**Table 3:** Multivariable analysis for patients after LRRT

Characteristic	Hazard ratio	95%CI	*P* value
**No. of metastatic organs**			
1	Reference		
> 1	2.177	1.445–3.281	< 0.001
**No. of metastatic lesions**			
≤ 5	Reference		
> 5	2.772	1.860–4.132	< 0.001
**EBV-DNA (Copies/ml)**			
≤ 25,000	Reference		
> 25,000	1.355	0.956–1.355	0.035
**LRRT technique**			
2DCRT	Reference		
IMRT	0.935	0.667–1.312	0.699

### Clinical characteristics of patients that did or did not underwent LRRT in different risk stratifications

According to the risk factors defined in the previous subsection, patients were divided into eight subgroups: group A, single organ metastasis, EBV DNA concentration ≤ 25,000 copies/ml, and 5 or fewer metastatic lesions; group B, single organ metastasis, EBV DNA concentration > 25,000 copies/ml, and 5 or fewer metastatic lesions; group C, multiple organs metastasis, EBV DNA concentration ≤ 25,000 copies/ml, and 5 or fewer metastatic lesions; group D, multiple organs metastasis, EBV DNA concentration > 25,000 copies/ml, and 5 or fewer metastatic lesions; group E, single organ metastasis, EBV DNA concentration ≤ 25,000 copies/ml, and more than 5 metastatic lesions; group F, single organ metastasis, EBV DNA concentration > 25,000 copies/ml, and more than 5 metastatic lesions; group G, multiple organs metastasis, EBV DNA concentration ≤ 25,000 copies/ml, and more than 5 metastatic lesions; and group H, multiple organs metastasis, EBV DNA concentration > 25,000 copies/ml, and more than 5 metastatic lesions.

**Fig. 4:**
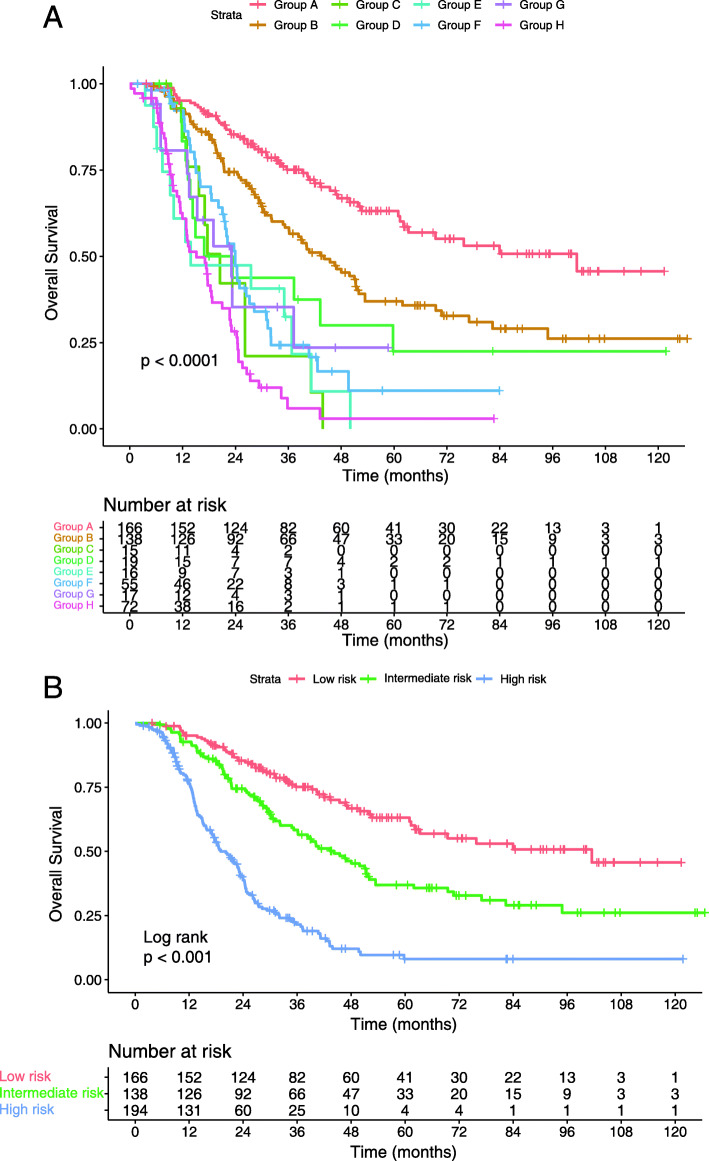
Kaplan–Meier survival curves for the risk factors (**a**) and different risk stratifications (**b**)

The Kaplan-Meier survival curves showed that patients in groups C-H had shorter OS than those in groups A-B; moreover, the OS of group A was significantly longer than that of group B (*p* < 0.05 for all). However, further paired comparisons revealed no significant differences in OS among groups C-H (*p* > 0.05 for all) (Fig. 4a). Subsequently, group A was classified as a low-risk subgroup (single organ metastasis, EBV DNA concentration ≤ 25,000 copies/ml, and 5 or fewer metastatic lesions), group B was classified as an intermediate-risk subgroup (single organ metastasis, EBV DNA concentration > 25,000 copies/ml, and 5 or fewer metastatic lesions), and groups C-H were classified as a high-risk subgroup (multiple organs metastasis or more than 5 metastatic lesions or both). The survival curves of patients in different risk strata are displayed in Fig. 4b. According to the Pearson χ^2^ test, the subgroups only differed in chemotherapy regimens (*p* < 0.001, *p* = 0.004 in low-risk and high-risk subgroups respectively) (Table 4) and no significant difference was found in other clinical characteristics.

**Table 4:** The clinical characteristics of the patients that did RT and did not do RT in different risk stratifications

	Low risk			Intermediate risk			High risk		
Characteristic	non-LRRT	LRRT	*P* value	non-LRRT	LRRT	*P* value	non-LRRT	LRRT	*P* value
**Age (years)**									
≤ 47	15 (44.1%)	69 (52.3%)	0.445	20 (41.7%)	42 (46.7%)	0.595	52 (49.5%)	48 (53.5%)	0.567
> 47	19 (55.9%)	63 (47.7%)		28 (58.3%)	48 (53.3%)		53 (50.5%)	41 (46.1%)	
**Gender**									
Male	28 (82.4%)	110 (83.3%)	1.000	34 (70.8%)	75 (83.3%)	0.124	93 (88.6%)	74 (83.1%)	0.304
Female	6 (17.6%)	22 (16.7%)		14 (29.2%)	15 (16.7%)		12 (11.4%)	15 (16.9%)	
**Smoking**									
No smoking	22 (64.7%)	70 (53.0%)	0.250	28 (58.3%)	53 (58.9%)	1.000	59 (56.2%)	46 (51.7%)	0.565
Smoking	12 (35.3%)	62 (47.0%)		20 (41.7%)	37 (41.1%)		46 (43.8%)	43 (48.3%)	
**Family history**									
No	31 (91.2%)	113 (85.6%)	0.425	44 (91.7%)	83 (92.2%)	1.000	95 (90.5%)	81 (91.0%)	1.000
Yes	3 (8.8%)	19 (14.4%)		4 (8.3%)	7 (7.8%)		10 (9.5%)	8 (9.0%)	
**T stage #**									
T_1_-T_2_	4 (11.8%)	24 (18.2%)	0.451	9 (18.8%)	17 (18.9%)	1.000	16 (15.2%)	13 (14.6%)	1.000
T_3_-T_4_	30 (88.2%)	108 (81.8%)		39 (81.3%)	73 (81.1%)		89 (84.8%)	76 (85.4%)	
**N stage #**									
N_0_-N_1_	9 (26.5%)	35 (26.5)	1.000	6 (12.5%)	18 (20.0%)	0.348	15 (14.3%)	20 (22.5%)	0.189
N_2_-N_3_	25 (73.5%)	97 (73.5%)		42 (87.5%)	72 (80.0%)		90 (85.7%)	69 (77.5%)	
**No. of metastatic organ**									
1	34 (100.0%)	132 (100.0%)	–	48 (100.0%)	90 (100.0%)	**–**	34 (32.4)	37 (41.6%)	0.232
> 1	–	–		–	–		71 (67.6%)	52 (58.4%)	
**No. of metastatic tumor number**									
≤ 5	34 (100.0%)	132 (100.0%)	–	48 (100.0%)	90 (100.0%)	**–**	15 (14.3%)	19 (21.3%)	0.256
> 5	–	–		–	–		90 (85.7%)	70 (78.7%)	
**EBV-DNA (Copies/ml)**									
≤ 25,000	34 (100.0%)	132 (100.0%)	–	–	–	–	25 (23.8%)	23 (25.8%)	0.868
> 25,000	–	–		48 (100.0%)	90 (100.0%)		80 (76.2%)	66 (74.2%)	
**Chemotherapy regimens**									
TPF	7 (20.6%)	36 (27.3%)	< 0.001	11 (22.9%)	26 (28.9%)	0.336	22 (21.0%)	26 (29.2%)	0.004
TP	5 (14.7%)	36 (27.3%)		10 (20.8%)	20 (22.2%)		21 (20.0%)	29 (32.6%)	
PF	15 (44.1%)	27 (20.5%)		10 (20.8%)	26 (28.9%)		33 (31.4%)	18 (20.2%)	
GP	5 (14.7%)	3 (2.3%)		4 (8.3%)	6 (6.7%)		9 (8.6%)	0 (0.0%)	
Others	2 (5.9%)	30 (22.7%)		13 (27.1%)	12 (13.3%)		20 (19.0%)	16 (18.0%)	

### Patients’ outcomes in different risk stratifications

**Fig. 5:**
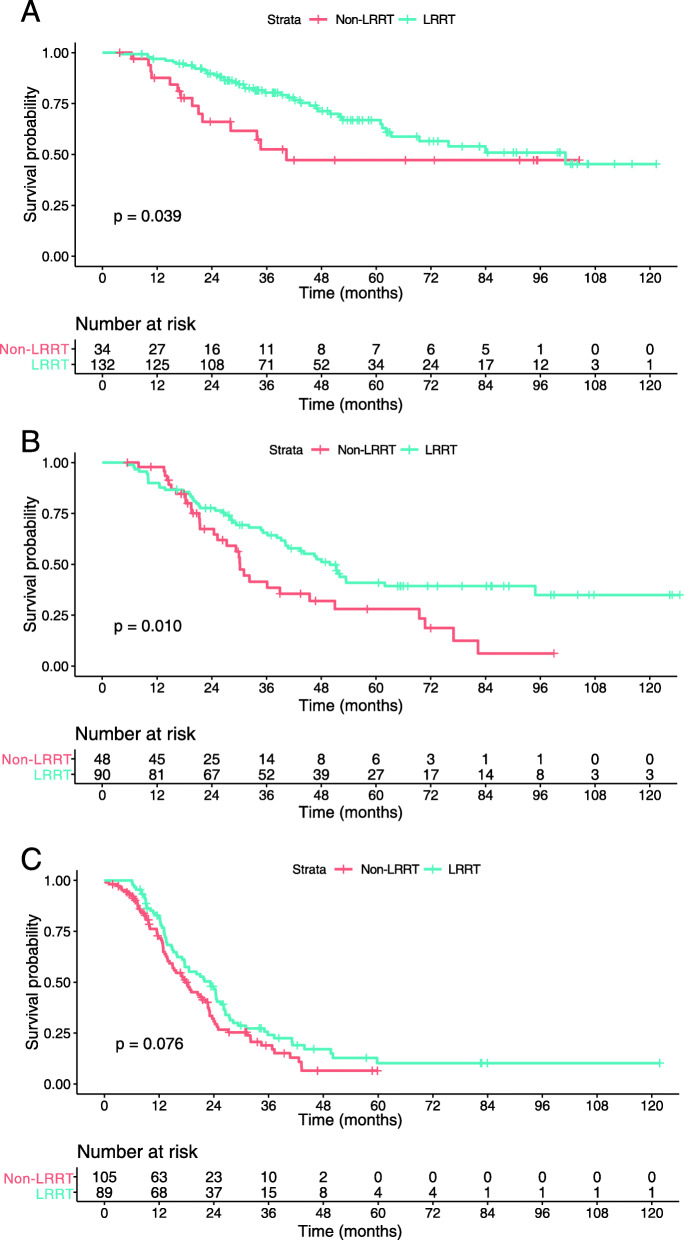
Comparison of overall survival of patients in the locoregional radiotherapy (LRRT) and non-LRRT groups: low-risk patients (**a**), intermediate-risk patients (**b**) and high-risk patients (**c**)

The differences in OS between patients that did and did not receive LRRT in each classification of risk were further investigated in this study. Interestingly, not all patients benefited from LRRT. Statistical differences were found in OS among patients in the low-risk and intermediate-risk subgroups (*p* = 0.039 and *p* = 0.010, respectively), whereas no significant difference was found in the high-risk subgroup (*p* = 0.076) (Fig. 5). Subsequently, Cox proportional hazards regression model was performed for all subgroups (Table 5) and it was found that LRRT lowered the mortality risk for patients in the low-risk (HR, 0.490; 95% CI, 0.232–0.960; *p* = 0.042) and intermediate-risk subgroups (HR, 0.582; 95% CI, 0.357–0.947; *p* = 0.029); however, it did not affect high-risk patients (HR, 0.718; 95% CI, 0.499–1.033; *p* = 0.074). Regarding these patients, the mortality risk was higher for those who had multiple organs metastasis (HR, 1.518; 95% CI, 1.032–2.234; *p* = 0.034), whereas the presence of multiple (> 5) metastatic lesions or a pretreatment EBV DNA copies level above cutoff did not seem to worsen this risk (HR: 1.564, 95% CI: 0.955–2.562, *p* = 0.076; HR: 1.127, 95% CI: 0.745–1.707, *p* = 0.571, respectively).

**Table 5:** Multivariable analysis for patients prognosis in different risk stratifications

	Low risk			Intermediate risk			High risk		
Characteristic	HR	95%CI	*P* value	HR	95%CI	*P* value	HR	95%CI	*P* value
**Age (years)**									
≤ 47	Reference			Reference			Reference		
> 47	1.311	0.724–2.375	0.371	1.403	0.870–2.262	0.165	1.051	0.745–1.480	0.778
**Gender**									
Male	Reference			Reference			Reference		
Female	0.486	0211–1.118	0.089	0.657	0.361–1.194	0.168	1.527	0.948–2.461	0.082
**Smoking**									
No smoking	Reference			Reference			Reference		
Smoking	1.219	0.665–2.235	0.522	1.130	0.693–1.843	0.632	1.266	0.883–1.816	0.199
**Family history**									
No	Reference			Reference			Reference		
Yes	0.748	0.246–2.278	0.609	0.462	0.157–1.355	0.160	1.057	0.572–1.955	0.859
**T stage**									
T_1_-T_2_	Reference			Reference			Reference		
T_3_-T_4_	0.723	0.349–1.499	0.383	1.110	0.605–2.036	0.737	0.843	0.526–1.349	0.475
**N stage**									
N_0_-N_1_	Reference			Reference			Reference		
N_2_-N_3_	2.318	1.098–4.891	0.027	1.051	0.589–1.875	0.867	1.367	0.860–2.175	0.186
**No. of metastatic organs**									
1	–			–			Reference		
> 1	–	–	–	–	–	–	1.518	1.032–2.234	0.034
**No. of metastatic tumor**									
≤ 5	–			–			Reference		
> 5	–	–	–	–	–	–	1.564	0.955–2.562	0.076
**EBV-DNA (Copies/ml)**									
≤ 25,000	–			–			Reference		
> 25,000	–	–	–	–	–	–	1.127	0.745–1.707	0.571
**Chemotherapy regimens**									
TPF	Reference			Reference			Reference		
TP	0.717	0.294–1.749	0.465	0.770	0.386–1.537	0.459	0.818	0.500–1.341	0.426
PF	0.814	0.336–1.971	0.648	0.619	0.295–1.300	0.205	0.962	0.584–1.583	0.878
GP	0.922	0.379–2.241	0.858	0.925	0.486–1.759	0.812	0.700	0.417–1.174	0.176
Others	1.696	0.439–6.550	0.444	0.859	0.316–2.333	0.766	0.688	0.258–1.837	0.455
**LRRT**									
Non-LRRT	Reference			Reference			Reference		
LRRT	0.490	0.232–0.960	0.042	0.582	0.357–0.947	0.029	0.718	0.499–1.033	0.074

## Discussion

In this study, dmNPC patients were stratified into different risk levels based on the number of metastatic lesions, number of metastatic organs, and level of pretreatment EBV DNA. In exploring the role of LRRT, the study found that patients with a single metastatic organ and no more than five metastases benefited the most from the therapy, and among which, the patients with EBV DNA concentration ≤ 25,000 copies/ml have better OS than those with EBV DNA concentration > 25,000 copies/ml, which provides important information for individual treatment management in clinical practice.

Distant metastasis has become the main cause of death for NPC patients [3, 10]. Among all patients with distant metastasis, some had it detected at initial admission, which is defined as dmNPC [5]. Unlike patients with metastasis after treatment, these patients had no previous LRRT for the primary tumor. Whether the use of LRRT is necessary has become a concern of clinicians. Recently, two studies have shown that the addition of LRRT to PCT is associated with a longer survival time for dmNPC [6, 11]. However, another question needs to be clarified: can all these patients benefit from LRRT? By analyzing the role of LRRT in dmNPC, You et al. found that patients with liver metastasis did not benefit from the primary tumor treatment while patients with other metastasis did [12]. But the M1 stage subdivisions of the study did not take tumor burden and pre-treatment EBV DNA copies into considerations. Similarly, NCCN guidelines recommend chemotherapy combined with LRRT only for patients with limited metastasis sites or a low tumor burden, but the standard for “low tumor burden” of dmNPC is not defined [7].

In this study, LRRT can improve OS and reduce risk of death for dmNPC patients. Compared with 2DCRT, IMRT might neither improve OS of dmNPC patients nor reduce risks of death of dmNPC patients, which was different from locally advanced NPC. Consistently with previous studies, more than five metastases and multiple metastatic organs were identified as independent risk factors [13, 14]. The cutoff value for the number of metastases was based on the definition of ‘oligo metastasis’ used in clinical trials [15]. Moreover, it has been demonstrated that pretreatment EBV DNA is closely associated with prognosis in locally advanced NPC [16, 17]. Similar to non-metastatic patients, this study suggested that high levels of EBV DNA may also be associated with worse prognosis in dmNPC patients. Based on the three identified prognostic factors, all patients were divided into eight groups then further classified these groups into three risk levels according to the statistical differences in survival among the eight groups. Due to limitations of the TNM staging system for metastatic patients, the prognosis of patients in the M1 stage could not be further classified. Based on the results of this study, these patients were stratified into different risk levels to facilitate a general prognosis assessment according to their baseline data.

More importantly, this study identified the optimal candidates for LRRT based on a biomarker and tumor burden. In exploring the role of LRRT, it was found that only low- and moderate-risk patients (single metastatic organ and no more than five metastases) benefited from primary tumor treatment. This phenomenon may be explained by the following: as the illness of patients with oligo metastases is more likely to be controlled by PCT, they should be responsive to LRRT, which is a good way to prevent further disease progression from the primary tumor. However, primary LRRT may not improve the survival of patients with more than five metastatic lesions or multiple metastatic organs. Considering the high cost and serious treatment-related toxicity, the administration of LRRT should be treated with caution. The main treatment goals for high-risk patients should include long-term survival with tumor and improvement of life quality [18]. Therefore, systemic chemotherapy and symptomatic treatment may be preferred treatment strategies for these patients.

The continuous progress of medical science has improved the OS of dmNPC patients remarkably. However, as shown in this study, the survival condition of high-risk patients is still unsatisfactory, with a 3-year survival rate of 35%. Therefore, new therapeutic methods need to be developed, such as epidermal growth factor receptor (EGFR) targeted drugs. Unfortunately, although EGFR overexpression was detected in NPC, a retrospective study showed that the use of anti-EGFR drugs did not further improve the survival of dmNPC patients [19, 20]. Immunotherapy, represented by PD-1 antibodies treatment, is another recent promising research path [21]. To explore the treatment efficacy of PD-1 antibody in metastatic NPC, our group launched a global multicenter, double-blind, randomized controlled phase III clinical trial. At present, patients’ enrollment has been completed and we are looking forward to the results of long-term follow-up.

The current study has the following limitations: it is a retrospective study, therefore, selective bias was unavoidable and different imaging methods might have effects on evaluations of tumor burden. Furthermore, this study conducted a single center study and most cases came from epidemic areas. Therefore, our conclusions need to be confirmed by multicenter prospective clinical trials.

## Supplementary Information

**Additional file 1: Figure S1.** ROC curve analysis used to determine the cutoff value of pretreatment EBV DNA levels.

## Data Availability

The datasets used and/or analyzed during the current study are available from the corresponding author on reasonable request. If anyone wants to request the data from the study, please contact maihq@sysucc.org.cn.
