# Supplementary material for: Subdivision of de-novo metastatic nasopharyngeal carcinoma based on tumor burden and pretreatment EBV DNA for therapeutic guidance of locoregional radiotherapy
Source: BMC Cancer. 2021 May 11;21:534. doi: 10.1186/s12885-021-08246-0 (PMC8111972; doi:10.1186/s12885-021-08246-0)

Figure S1: ROC curve analysis used to determine the cutoff value of pretreatment EBV DNA levels.

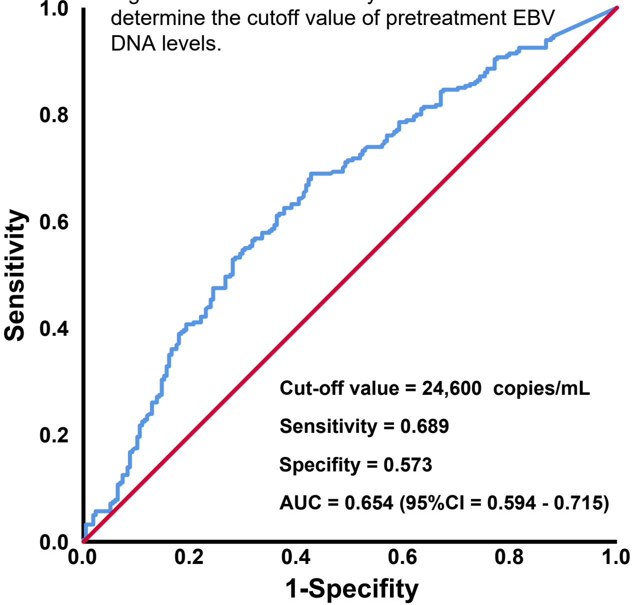

Supplement: Supplementary file 1 — Additional file 1: Figure S1. ROC curve analysis used to determine the cutoff value of pretreatment EBV DNA levels. [file 12885_2021_8246_MOESM1_ESM.pdf]
